# Performance Comparison of Junior Residents and ChatGPT in the Objective Structured Clinical Examination (OSCE) for Medical History Taking and Documentation of Medical Records: Development and Usability Study

**DOI:** 10.2196/59902

**Published:** 2024-11-21

**Authors:** Ting-Yun Huang, Pei Hsing Hsieh, Yung-Chun Chang

**Affiliations:** 1 Shuang-Ho Hospital, Taipei Medical University, New Taipei City, Taiwan; 2 Graduate Institute of Data Science, Taipei Medical University, Zhonghe District, New Taipei City, Taiwan

**Keywords:** large language model, medical history taking, clinical documentation, simulation-based evaluation, OSCE standards, LLM

## Abstract

**Background:**

This study explores the cutting-edge abilities of large language models (LLMs) such as ChatGPT in medical history taking and medical record documentation, with a focus on their practical effectiveness in clinical settings—an area vital for the progress of medical artificial intelligence.

**Objective:**

Our aim was to assess the capability of ChatGPT versions 3.5 and 4.0 in performing medical history taking and medical record documentation in simulated clinical environments. The study compared the performance of nonmedical individuals using ChatGPT with that of junior medical residents.

**Methods:**

A simulation involving standardized patients was designed to mimic authentic medical history–taking interactions. Five nonmedical participants used ChatGPT versions 3.5 and 4.0 to conduct medical histories and document medical records, mirroring the tasks performed by 5 junior residents in identical scenarios. A total of 10 diverse scenarios were examined.

**Results:**

Evaluation of the medical documentation created by laypersons with ChatGPT assistance and those created by junior residents was conducted by 2 senior emergency physicians using audio recordings and the final medical records. The assessment used the Objective Structured Clinical Examination benchmarks in Taiwan as a reference. ChatGPT-4.0 exhibited substantial enhancements over its predecessor and met or exceeded the performance of human counterparts in terms of both checklist and global assessment scores. Although the overall quality of human consultations remained higher, ChatGPT-4.0’s proficiency in medical documentation was notably promising.

**Conclusions:**

The performance of ChatGPT 4.0 was on par with that of human participants in Objective Structured Clinical Examination evaluations, signifying its potential in medical history and medical record documentation. Despite this, the superiority of human consultations in terms of quality was evident. The study underscores both the promise and the current limitations of LLMs in the realm of clinical practice.

## Introduction

Large language model (LLM) chatbots have shown great potential in producing human-like conversations and have already been applied in several sectors [1,2]. Recent research in medical fields has consistently demonstrated the effectiveness of LLMs [3-5], particularly in comparison with human counterparts. Watari et al [6] remarkably found that ChatGPT outperformed medical residents in the general medicine in-training examination, showcasing the advanced capabilities of LLMs in understanding and applying medical knowledge [7]. ChatGPT also demonstrated a significant advantage over medical students in areas of clinical reasoning and medical record keeping [8]. A key area of this superiority was particularly noted in the creation of problem lists, a critical component in patient care and diagnosis [9,10]. These findings collectively underscore the potential of artificial intelligence (AI) tools such as ChatGPT in both medical education and clinical practice [11-13].

While previous studies have demonstrated the efficacy of ChatGPT applied in medical situations [14,15], this research predominantly relied on preconstructed narrative inputs rather than real-time conversational interactions. This methodological approach raises questions about ChatGPT capabilities in actual clinical settings, where dynamic conversation is a critical component of patient history taking. To address this research gap, our team applied the Objective Structured Clinical Examination (OSCE) methodology to simulate the clinical setting. OSCEs, a pivotal educational tool first described by Harden and Gleeson [16], are renowned for their effectiveness in assessing the clinical skills of medical trainees within a structured and controlled setting. Standardized patients (SPs) and participants with no medical background using ChatGPT in the role of clinicians were incorporated in our simulation, which provides an ideal platform to conduct a comparative analysis of the clinical consultation competencies between clinicians and ChatGPT. We further evaluated the OSCE scores to measure the efficacy of AI in emulating human-like clinical reasoning and decision-making skills. The quality of the medical records generated by AI was also compared with the quality of those produced by human trainees.

Our study aims to explore whether ChatGPT can effectively engage in real-time conversations with patients for medical history taking and medical record documentation. This research seeks to address the current gap in the literature concerning the capabilities of ChatGPT in real-time patient interactions. Our research question is: “Can ChatGPT perform medical history taking and medical record documentation as effectively as junior medical residents in a simulated clinical environment?”

## Methods

### Using the OSCE Framework for Comparative Analysis

Our study used an OSCE format, comprising 10 distinct scenarios, which were used to assess junior residents or medical students in previous examinations. The rubric and cases for the OSCE were included in Multimedia Appendix 1. The tasks and history-taking scenarios for the OSCE were selected based on the guidelines of the national OSCE examination. These were developed through expert consensus using the Delphi method by 5 experts, focusing on each question and evaluation item. The tasks included taking medical histories for different diseases, explaining medical conditions, and providing medical consultations. These tasks had been tested multiple times in previous settings, with some even adopted in official national medical licensing examinations, ensuring their relevance and robustness.

Each scenario was allocated a duration of 10 minutes, within which participants were tasked with obtaining a medical history from SPs and filling out a medical record. To maintain the rigidity of the examination structure, an alarm was set to sound 1 minute prior to the transition to the next station, signaling the end of the current patient interaction.

### Participants

The study enrolled 5 SPs, each of whom was randomly assigned 2 scripts. These SPs were instructed to respond to questions strictly based on their assigned script and to refrain from improvising answers to unscripted questions. Crucially, SPs were kept unaware of the participants’ identity, who were either actual clinicians or nonmedical individuals using ChatGPT for assistance.

Five junior residents and 5 laypersons were recruited. Since our intention is to evaluate the potential assistance from ChatGPT for junior residents in taking patient history and writing medical records, the junior residents in our study are in postgraduate years 1 and 2 and as well as in the first year of their residency in emergency medicine. After they passed the medical licensing examinations, they no longer receive any OSCE training, so the number of times they undergo OSCE training is the same. All laypersons in our study were graduate students from the computer science institute and did not have any medical training background. Therefore, they are familiar with inputting information into a computer and can understand the output from ChatGPT to deliver the message to our SPs effectively. Calculating the effective sample size in our study design is challenging. Given that each scenario takes 10 minutes, and considering the need to avoid attention fatigue, we determined that a total of 10 scenarios approximately 100 minutes per resident or layperson is appropriate.

The junior residents were instructed to approach the OSCE as per their medical training and apply their clinical skills and knowledge. In contrast, the nonmedical participants were directed to use ChatGPT for generating questions and interpreting responses from the SPs. They were explicitly instructed to limit their queries and responses to those formulated by ChatGPT, thereby ensuring a consistent methodology across all nonmedical participants. In addition, unlike traditional OSCEs, participants in this study were required to simultaneously conduct patient interviews and document medical records. Typically, after asking each question, the interviewer would enter the information into the medical record. This method of patient interviewing, commonly practiced in our research team’s country, requires medical participants to input medical records into the computer after each question. For nonmedical participants, they had to input each question into ChatGPT using the computer. The computer screen was positioned in such a way that the SPs could not see it, preventing them from discerning whether the participants’ use of computer was for inputting information for themselves or for using ChatGPT.

In our study, 2 iterations of ChatGPT, versions 3.5 and 4.0, were used. Initially, we intended to use ChatGPT-4.0 only; however, the usage cap for 1 subscribed individual user is 40 messages per 3 hours with GPT-4T. Since we exceeded this limit during our study, we had to switch to using GPT-3 instead. Therefore, a total of 13 interviews were conducted using this version toward the end of our study. The other 37 interviews were carried out using ChatGPT 3.5. Throughout the study, the participants used a set of standardized prompts during the study to ensure consistency across all interactions, with no room for improvisation or adaptation as all prompts were preset. There was no prompt chaining involved, and the temperature setting of the chatbot was not adjusted; default settings were used throughout the study to maintain uniformity in the responses generated by ChatGPT. There was no use of prompt chaining, and the temperature setting for ChatGPT was kept at the default level to maintain uniformity in the generated responses throughout the study.

### Evaluation of Medical History Taking and Medical Record–Writing Abilities

The OSCE global score and checklist score used to evaluate the examinees’ performance were standardized scale developed and reviewed by experienced medical experts. including the specific tasks. The OSCE Global Score often reflects elements such as communication skills, professional behavior, clinical reasoning, and the ability to integrate and apply clinical knowledge in a practical setting. The total score is 5 points. On the other hand, the OSCE Checklist Score is a more objective measure and usually consists of a series of specific tasks or objectives, such as performing a specific examination technique or asking the right questions; in this way it provides a detailed assessment of their technical and procedural abilities. Taking clinical reasoning, for example, if the doctor could name at least 3 differential diagnoses, the score was 3; for 2 differential diagnoses, the score was 2; and for 1 or no differential diagnoses, the score was 1. The total score of each task is 20‐24 points.

The quality of patient interview and medical records produced by the participants was assessed using a comprehensive 5-point Likert scale to cover 5 main aspects, which resulted in a total score of 25. This assessment specifically focused on evaluating the reasoning, completeness of the medical record, precision, accuracy, and grammatical correctness of the documentation. Two independent physicians evaluated the quality of the case documentation and patient interviews by checking the audio recordings and medical records. Prior to the assessment, these physicians were unaware of which medical records were documented by junior residents and which were generated by ChatGPT.

Moreover, the SPs played a crucial role in evaluating the quality of the interviews. They answered a total of 5 questions with a 5-point Likert scale to assess the following dimensions of the “physician’s” performance: sufficient professional knowledge, clear explanations for assessments, appropriately addressing patients’ concerns, effective communication skills, and humanized care. The junior residents were tasked with reviewing and evaluating both their own medical records and those generated by ChatGPT after OSCE. This review process was conducted using a 5-point Likert scale that was designed to provide a detailed assessment of various key aspects, including the overall quality of the medical records, the potential assistance provided by ChatGPT in medical record documentation, and the accuracy of the differential diagnoses recorded.

### Statistical Analysis

In our analysis, we used the Mann-Whitney *U* test to assess differences in performance between human participants and various iterations of ChatGPT. We made this choice due to the characteristics of our data, specifically the small sample size and the absence of a normal distribution. The Mann-Whitney *U* test, a nonparametric method, was particularly suited for our needs as it compares median values and IQRs, thus accommodating data that do not conform to normality. For the statistical comparisons, we applied a 2-tailed test approach, and results yielding a *P* value of <.05 were deemed statistically significant, indicating meaningful differences in the performance metrics across groups.

### Ethical Considerations

This study was conducted with approval from the Taipei Medical University-joint institutional review board (TMU-JIRB; protocol N202307058, valid from August 17, 2023, through August 16, 2024). The TMU-JIRB granted a waiver of written informed consent due to the study’s design using standardized patients in a simulated environment. Verbal consent was obtained from all participants. All collected data, including audio recordings and medical documentation, were anonymized with identifiable information removed, and access was restricted to authorized research team members only. Standardized patients received appropriate compensation according to institutional guidelines, while junior residents and nonmedical participants volunteered without financial compensation, consistent with the educational nature of the study. The research strictly adhered to the ethical guidelines for human subject research as outlined by the TMU-JIRB.

## Results

### OSCE Checklist and Global Scale Scores

**Figure 1.**
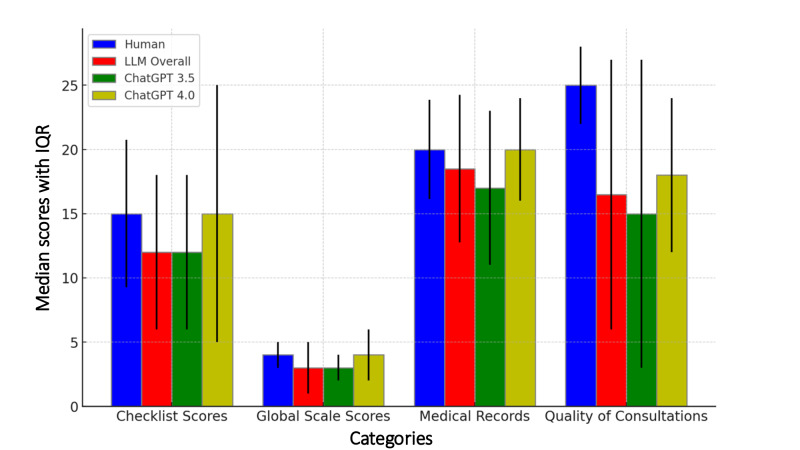
Comparative analysis on performance on the Objective Structured Clinical Examination, quality of medical records, and patient interview. LLM: large language model.

In the OSCE checklist evaluations, there were 50 test results from junior residents, 13 from ChatGPT 4.0, and 37 from ChatGPT 3.5. Clinicians achieved a median score of 15 (9.25-20.75), which was higher than the median score of the LLMs overall at 12 (IQR 6-18; *P*<.05). ChatGPT 4.0 performed better with a median score of 15 (IQR 5-25), showing no significant difference compared with humans (*P*=.28). A similar result was observed in the OSCE global scale scores, where humans outperformed the LLMs overall (median of 4 vs 3; *P*<.05). ChatGPT 4.0 scored higher with a median of 4, which was again statistically nonsignificant compared with humans (*P*=.15). For medical record scores, clinicians scored higher on medical record quality with a median of 20 (IQR 16.125-23.875), compared with LLMs at 18.5 (IQR 12.75-24.25; *P*<.05). ChatGPT 4.0 scored similarly to humans with a median of 20 (IQR 16-24), but this difference was not statistically significant (*P*=.61; Table 1 and Figure 1).

**Figure 2.**
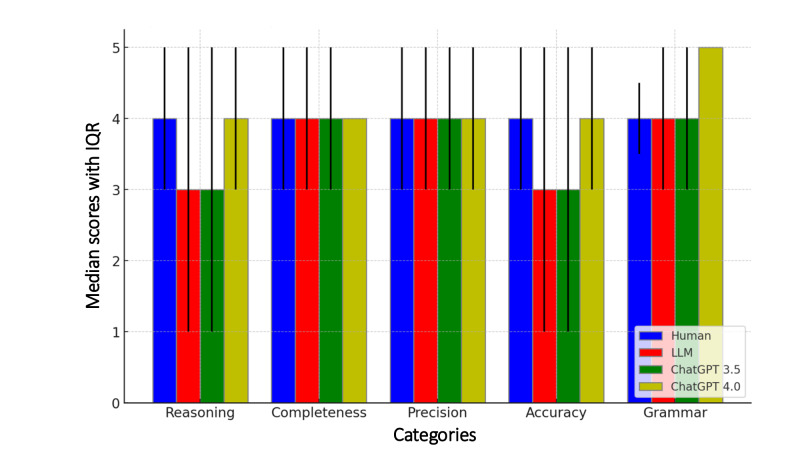
Comparative analysis on 5 metrics of medical documentation with IQR. LLM: large language model.

In a detailed comparison across reasoning, completeness of medical records, description precision, diagnosis accuracy, and grammatical correctness of the documentation, LLM was comparable and sometimes surpassed the human clinicians (Figure 2). Specifically, ChatGPT nearly matched clinicians in reasoning, particularly version 4.0, and for completeness and description precision, ChatGPT was comparable, except that version 3.5 showed lower scores. ChatGPT noticeably lagged in diagnosis accuracy yet surpassed clinicians in grammar.

**Table 1.** Comparative analysis of the overall performance of human and LLM.

Category	Human, median (IQR)	LLM^a^ overall, median (IQR)	*P* value	ChatGPT-3.5, median (IQR)	*P* value	ChatGPT-4.0, median (IQR)	*P* value
Checklist scores	15(9.25-20.75)	12(6-18)	<.05	12(6-18)	<.05	15(5-25)	.28
Global scale scores	4(3-5)	3(1-5)	<.05	3(2-4)	<.05	4(2-6)	.15
Medical records	20(16.125-23.875)	18.5(12.75-24.25)	<.05	17(11-23)	<.05	20(16-24)	.61
Quality of consultations	25(22-28)	16.5(6-27)	<.05	15(3-27)	<.05	18(12-24)	<.05

a LLM: large language model.

### Quality of Consultations

**Figure 3.**
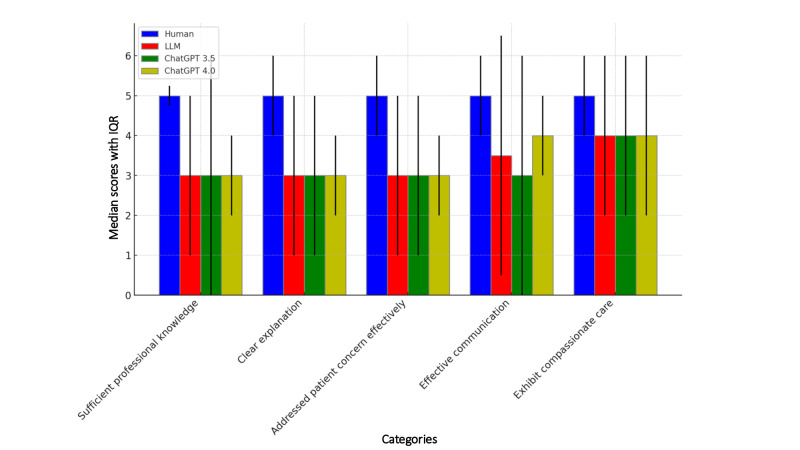
Comparative analysis on 5 metrics of consultation quality with IQR. LLM: large language model.

The feedback from the SPs showed that humans excelled over LLMs in overall interview quality, including professional knowledge, clear explanations, responsiveness to concerns and worries, effective communication skills, and compassionate care (Table 1 and Figure 3). Human clinicians achieved a significantly higher median score of 25 (IQR 22-28) compared to LLMs overall at 16.5 (IQR 6-27; *P*<.05), ChatGPT 3.5 at 15 (IQR 3-27; *P*<.05), and ChatGPT 4.0 at 18 (IQR 12-24; *P*<.05). In evaluating the 5 key aspects of quality consultation, clinicians’ scores in professional knowledge and clear explanations were significantly higher than those of the LLMs (both ChatGPT-3.5 and -4.0) in all categories (*P*<.05).

### Clinicians’ Feedback on ChatGPT

**Figure 4.**
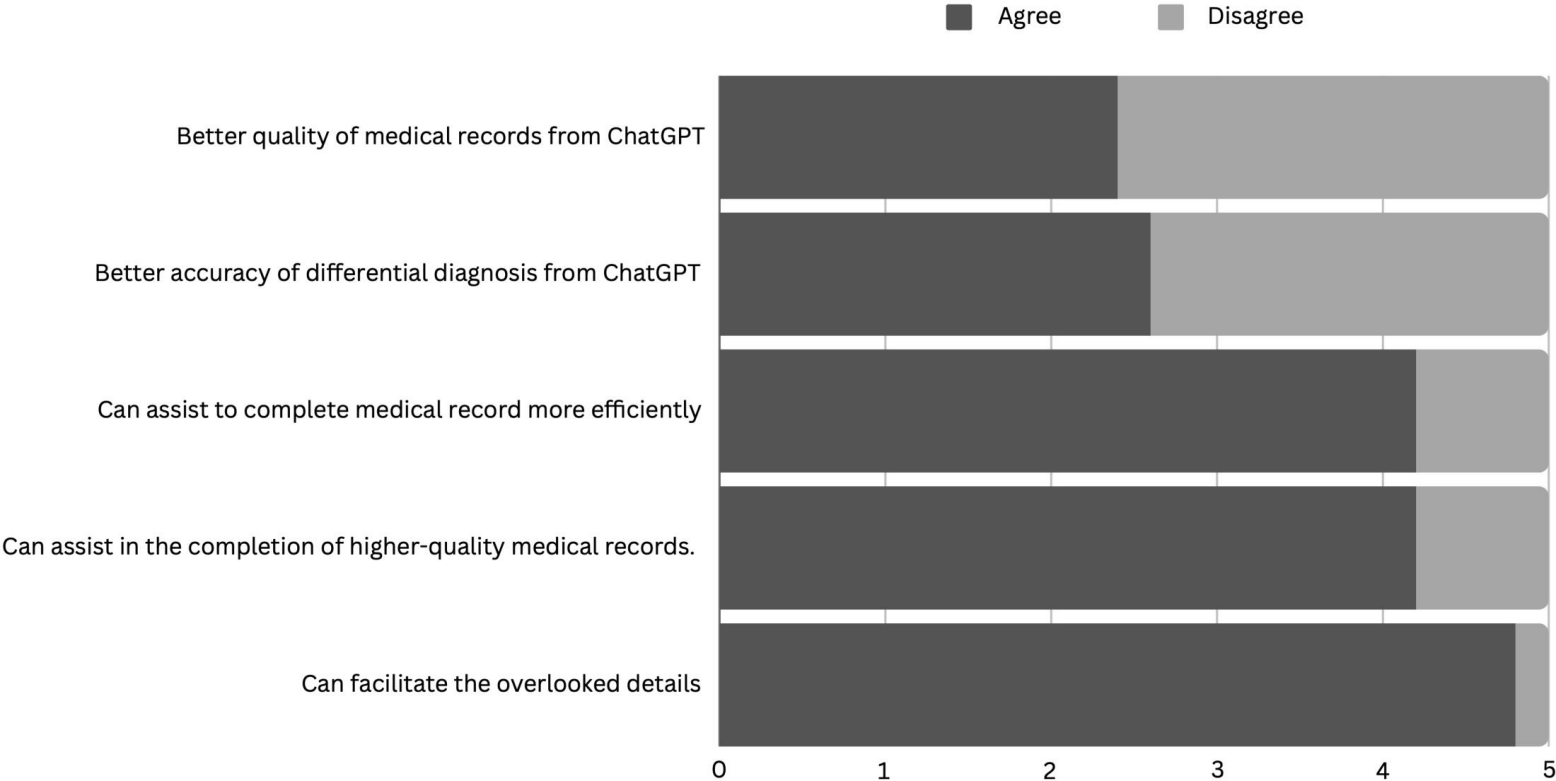
Clinician’s feedback on ChatGPT.

Medical professionals considered that their medical records were slightly better than those generated by ChatGPT (score 2.6/5), while the accuracy of differential diagnosis was deemed comparable (score 2.4/5). Nonetheless, they recognized the advantages of LLM in improving documentation speed and quality (score 4/5), and they especially appreciated ChatGPT’s proficiency in uncovering missed details during patient interviews (4.8/5; Figure 4).

## Discussion

### Principal Findings

To our knowledge, this study is the first one using SPs in OSCE setting to compare the performance of LLM and clinicians in interviewing patients and writing medical records. Since OSCE is a widely accepted method to evaluate clinical skills, our study design closely mimics real-world patient interviews. In our study, ChatGPT demonstrated the capability to pass the OSCE examination under the criteria of Taiwan’s medical education system. Notably, ChatGPT 4.0 surpassed the clinicians in terms of OSCE checklist scores, global scale scores, and medical record quality, although these differences did not reach statistical significance.

In patient interviews, clinicians significantly outperformed ChatGPT, as evidenced by both subjective feedback from SPs and objective observations from 2 senior clinicians. Despite ChatGPT being trained on an extensive and diverse textual data set and having the ability to theoretically provide ample knowledge, it demonstrated shortcomings in clear, effective communication and compassionate care. Previous experiments have shown that while text-based assessments of empathy and professionalism are standard, translating these qualities into verbal and physical expressions can alter their perception. For instance, consider a ChatGPT response such as, “Given your symptoms, I would recommend a thorough examination and possibly some tests to ensure we address all potential concerns.” Although the response is professional and empathetic, participants with no medical background might not convey it with the necessary authority and confidence, thus reducing its perceived professionalism.

Therefore, if the text or questions generated by ChatGPT were directly communicated to the SPs without third-party interpretation, the results might differ. Further experiments are needed to confirm whether there is a significant difference between ChatGPT and humans in expressing empathy and professionalism in patient interviews. This limitation could lead to a disconnect in effectively relaying responses from ChatGPT to the SPs, thereby creating a gap in the delivery of appropriate medical knowledge during interactions. Moreover, ChatGPT’s training on a wide-ranging database can cause overgeneralization and result in responses that are too broad and less specific to certain medical contexts, unlike the focused expertise of clinicians. This highlights some areas where AI-assisted medical interactions can be improved [17-19]. These findings also suggest that the current LLMs still face challenges in fully replicating the nuanced judgment and empathy inherent in human clinical interactions. Moreover, the AI tool in this study was not specifically trained for OSCE scenarios. Customized training of AI systems to meet the unique requirements and subtleties of medical examinations such as OSCEs could further improve their performance. Such targeted training might enable AI to emulate the complex aspects of patient consultations more effectively and include empathy and more nuanced clinical judgments.

As for medical record documentation, ChatGPT shows promise for assistance, especially in enhancing grammar and completeness. These improvements would be vital for health care professionals for whom time efficiency and accuracy are crucial. However, in our observation, ChatGPT tends to omit “negative findings,” which are important for ruling out certain diagnosis and leading to precise reasoning. This observed omission in ChatGPT-generated medical records leads to deficient reasoning within the medical records and leads to a lower level of logical consistency compared with records by clinicians. In addition, recent research identifies “hallucinations” in clinical AI applications as a significant concern [20-22]. Our findings mirror this, with ChatGPT occasionally producing fabricated or irrelevant medical records [23-25]. Such inaccuracies underscore the need for human oversight and verification in using AI for clinical documentation to mitigate potential risks. Moreover, clinicians in our study perceived ChatGPT’s differential diagnosis accuracy as equal or slightly superior to their own, a finding not corroborated by senior clinicians’ observations. This discrepancy suggests that clinicians may struggle to discern the accuracy of ChatGPT’s information in ambiguous clinical situations.

### Limitations

The study faced several limitations and due to the usage limitations of ChatGPT-4.0, our study encountered a disparity in the number of interviews conducted between ChatGPT-4.0 and ChatGPT-3.5, which were 13 and 37, respectively. This discrepancy raises the need for further studies with enough interviews using ChatGPT-4.0 to comprehensively evaluate its performance in patient interviews and medical record documentation against that of clinicians. In addition, the small sample size of this study involved only 10 participants. Given the relatively small sample size, individual participant bias must be considered. Future studies should involve larger sample sizes and include participants with varying levels of clinical experience, ranging from junior residents to senior residents, attending physicians, and specialists from different fields, to minimize individual bias and provide a more comprehensive analysis.

Moreover, the nonmedical participants lacked formal training in clinical consultations, which potentially affected the quality of consultations as perceived by SPs. This absence of professional training in clinical demeanor and responsive skills might have biased the SPs’ assessments. For a more accurate replication of professional medical consultations, comprehensive training of responsive skills should be provided to nonmedical participants in future studies.

### Conclusions

Our study introduced an innovative approach to evaluate the performance of ChatGPT in comparison with junior medical residents, specifically in the context of real patient interactions. Operating within the framework of the OSCE, ChatGPT demonstrated the ability to meet the minimum requirements set by OSCE standards. While ChatGPT competency in patient consultation may not yet fully match that of junior residents, it shows considerable promise in the domain of medical record documentation. This aspect is crucial as it indicates ChatGPT’s potential utility in assisting with or streamlining the documentation process in clinical environments. However, the importance of human oversight and interaction remains paramount, especially in patient-facing scenarios where nuanced communication and empathy are essential.

Our study indicated that ChatGPT could become a valuable tool to assist in diagnosis and medical record writing. However, it is important to stress a key observation: while AI has previously demonstrated high standards in medical record quality, often exceeding human capabilities in past studies, its performance seemed to diminish when directly interacting with real humans during medical record generation. A significant risk associated with ChatGPT use in this context is the occurrence of “hallucinations,” where ChatGPT may generate incorrect or irrelevant information. This underscores the need for careful monitoring and verification of ChatGPT-generated medical records to ensure accuracy and reliability in clinical practice.

## Supplementary material

10.2196/59902 Multimedia Appendix 1 Guidelines and scoring criteria for clinical competency assessment.

## Abbreviations

AI: artificial intelligence
LLM: large language model
OSCE: Objective Structured Clinical Examination
SP: standardized patient
TMU-JIRB: Taipei Medical University-jJoint institutional review board
