# Supplementary material for: Performance Comparison of Junior Residents and ChatGPT in the Objective Structured Clinical Examination (OSCE) for Medical History Taking and Documentation of Medical Records: Development and Usability Study
Source: JMIR Med Educ. 2024 Nov 21;10:e59902. doi: 10.2196/59902 (PMC11612517; doi:10.2196/59902)
Supplement: Multimedia Appendix 1 [file mededu-v10-e59902-s001.docx]

**Candidate Guidelines**

Background Information

Ms. Hua He, a 40-year-old woman, attended a company health check and was found to have a low platelet count. She visited the clinic for help. She denies any history of chronic illness and is asymptomatic.

Test Topic

Focus on explaining the condition and its implications. Physical examinations and medical history inquiries are not scored.

Test Duration: 8 minutes

**Relevant Test Reports**

Blood Routine

WBC: 5,400/μL

Hemoglobin: 13.2 g/dL

MCV: 90 FL

Neutrophil: 64%

Lymphocyte: 32%

Monocyte: 2%

Platelet: 56,000/μL

Biochemistry Data: All within normal limits

**Scoring Sheet**

**Total Score: 20 points**

**Overall Score: ______ points**

**Test Items:**

□Medical History Inquiry

□Doctor-Patient Communication and Health Education

□Physical Examination

■Condition Explanation and Clinical Management

**Test Duration: 8 minutes**

Candidate Number:

**Scoring Criteria (10-15 items):**

| **Item** | **Not Asked (0)** | **Partially Asked (1)** | **Fully Asked (2)** | **Comments** |
| --- | --- | --- | --- | --- |
| 1. Self-introduction and addressing the patient |  |  |  |  |
| 2. Explanation of platelet production and function |  |  |  |  |
| 3. Explanation of thrombocytopenia symptoms (e.g., mucosal bleeding, subcutaneous bleeding, other symptoms) |  |  |  |  |
| 4. Explanation of possible reasons for low platelet count without clinical symptoms |  |  |  |  |
| 5. Explanation of possible reasons for false-positive results (technique, antibodies, quality control) |  |  |  |  |
| 6. Explanation of causes of thrombocytopenia (examples of diseases) |  |  |  |  |
| 7. Explanation of mechanisms of thrombocytopenia (decreased production, excessive consumption) |  |  |  |  |
| 8. Explanation of further examinations |  |  |  |  |
| 9. Explanation of precautions after returning home |  |  |  |  |
| 10. Explanation of follow-up visits |  |  |  |  |

| **Overall Performance** | illustrate | Poor: 1 point | Needs Improvement: 2 points | Average: 3 points | Good: 4 points | Excellent: 5 points |
| --- | --- | --- | --- | --- | --- | --- |
|  | Score |  |  |  |  |  |

**Evaluator's Signature:** |

**Examiner Guidelines**

**Test Items:**

- Medical History Inquiry
- Physical Examination
- Condition Explanation and Clinical Management
- Doctor-Patient Communication and Health Education

**Examiner Task Tips:**

1. The purpose of this exam is to ensure that medical graduates meet the minimum clinical competency standards.
2. Maintain a fair mindset while scoring, focusing on the task at hand.
3. Align the test goals with the Candidate and Standardized Patient Guidelines and the Scoring Table.
4. Thoroughly read the scoring items and descriptions.
5. Use the consensus reference video during the pre-exam scoring consensus period to practice scoring.

**Test Scenario: Outpatient Clinic**

- **Standardized Patient Basic Information**: Ms. Hua He, a 40-year-old woman, discovered to have low platelet count during a company health check, visiting the clinic for help. She has no chronic illness history and is asymptomatic but appears anxious.

**Condition Summary:**

- **Initial Posture**: Seated
- **Case Scenario and Main Complaint**: Ms. Hua He, a 40-year-old woman, discovered to have low platelet count during a company health check, visits the clinic for help. She has no chronic illness history and is asymptomatic. No abnormalities were found during your physical examination.

**Medical History Inquiry:**

1. **Main Clinical Symptoms**: None
2. **Present Illness History**: Low platelet count found a month ago during company health check, asymptomatic
3. **Past Medical History**: None
4. **Family History**: None
5. **Drug History**: None
6. **Other Medical History**: None

**Physical Examination:**

- No abnormalities found

**Clinical Diagnosis and Management Reference for This Case:**

1. **Differential Diagnosis**:
   - False positive
   - Decreased production:
     - Bone marrow dysplasia
     - Aplastic anemia
     - Myelofibrosis
     - Leukemia
     - Multiple myeloma
     - Drug-induced megakaryocyte damage
     - Viral infection-induced megakaryocyte damage
   - Excessive consumption:
     - Severe infection
     - Autoantibodies
     - Immune thrombocytopenia
     - Various autoimmune diseases
     - Post-viral infection antibodies
     - Drug-induced antibodies
     - Post-vaccination antibodies including COVID-19
     - Widespread vascular thrombosis
     - Splenomegaly
   - Other
2. **Relevant Tests and Management**:
   - Exclude the possibility of false positives by checking for poor blood draw techniques, antibodies in the blood causing platelet aggregation at room temperature, and poor laboratory quality control.
   - Obtain detailed recent health information, family history, drug history, and conduct a physical examination.
   - Perform further blood tests to exclude the above differential diagnoses.
   - If splenomegaly is suspected, confirm with imaging tests such as ultrasound.
   - If the initial doctor is not a specialist, refer the patient to a hematologist for confirmation.
   - If the hematologist suspects a bone marrow problem, conduct a bone marrow biopsy and pathological examination.

**Props and Equipment**: SP (Standardized Patient)

**Scoring Explanation:**

1. **Self-introduction and Addressing the Patient**
   - **Fully Achieved**: Successfully introduces self and addresses the patient by full name
   - **Partially Achieved**: Completes only one of the above tasks
   - **Not Achieved**: Does neither
2. **Explanation of Platelet Production and Function**
   - **Fully Achieved**: Correctly explains that platelets are produced in the bone marrow and their function in hemostasis
   - **Partially Achieved**: Correctly explains only one aspect
   - **Not Achieved**: Provides no explanation
3. **Explanation of Thrombocytopenia Symptoms**:
   1. Mucosal bleeding (e.g., eye conjunctiva, oral mucosa, nasal mucosa)
   2. Subcutaneous bleeding (e.g., purpura, bruises)
   3. Other symptoms (e.g., hematuria, hematochezia, menorrhagia, prolonged menstrual period)
   4. **Fully Achieved**: Explains at least one symptom from each of the three categories
   5. **Partially Achieved**: Explains at least one symptom from any two categories
   6. **Not Achieved**: Explains at most one symptom from any category, or provides no explanation
4. **Explanation of Possible Reasons for Low Platelet Count without Clinical Symptoms** (false positive, not low enough to cause bleeding, chronic condition):
   - **Fully Achieved**: Explains all three reasons
   - **Partially Achieved**: Explains 1-2 of the reasons
   - **Not Achieved**: Provides no explanation of the above possibilities
5. **Explanation of Possible Reasons for False Positive Results** (1. Poor blood draw technique, 2. Presence of antibodies in the blood causing platelet aggregation at room temperature but not in the body, 3. Poor laboratory quality control):
   - **Fully Achieved**: Explains all three reasons
   - **Partially Achieved**: Explains 1-2 of the reasons
   - **Not Achieved**: Provides no explanation of the above possibilities
6. **Explanation of Causes of Thrombocytopenia** (Examples of diseases: bone marrow dysplasia, aplastic anemia, myelofibrosis, leukemia, multiple myeloma, drug toxicity, viral infection, severe infection, autoantibodies (including immune thrombocytopenia, autoimmune diseases, post-viral infection, drug-induced, post-vaccination including COVID-19), widespread vascular thrombosis, splenomegaly):
   - **Fully Achieved**: Provides examples of at least three diseases
   - **Partially Achieved**: Provides examples of 1-2 diseases
   - **Not Achieved**: Provides no examples
7. **Explanation of Mechanisms of Thrombocytopenia** (Decreased production, excessive consumption):
   - **Fully Achieved**: Mentions both mechanisms
   - **Partially Achieved**: Mentions only one mechanism
   - **Not Achieved**: Does not mention any mechanisms
8. **Explanation of Further Examinations**:
   1. Physical examination (confirm no signs of bleeding and check for splenomegaly)
   2. Medical history inquiry (check for bleeding history, medication use, recent infections, family history)
   3. Blood tests (confirm false positive and partial differential diagnosis)
   4. Bone marrow examination if needed
   5. **Fully Achieved**: Explains all four types of further examinations
   6. **Partially Achieved**: Explains 2-3 types of further examinations
   7. **Not Achieved**: Explains at most one type or provides no explanation
9. **Explanation of Post-Discharge Precautions**:
   1. Skin red spots, bruising, or purpura
   2. Eye conjunctiva, oral mucosa, gum bleeding, or nosebleeds that do not stop
   3. Hematuria or hematochezia
   4. Prolonged menstrual period and heavy bleeding
   5. Abnormal headaches
   6. **Fully Achieved**: Explains at least three of the above precautions
   7. **Partially Achieved**: Explains 1-2 of the above precautions
   8. **Not Achieved**: Provides no explanation
10. **Explanation of Follow-Up Appointments**:

1.Referral to hematology or hematology-oncology clinic

2.Immediate visit to the emergency room for severe symptoms

- **Fully Achieved**: Explains both points
- **Partially Achieved**: Explains only one point
- **Not Achieved**: Does not provide any recommendations
